# A taxonomy of demand-driven questions for use by evidence producers, intermediaries and decision-makers: results from a cross-sectional survey

**DOI:** 10.1186/s12961-024-01160-4

**Published:** 2024-07-05

**Authors:** Cristián Mansilla, Arthur Sweetman, Gordon Guyatt, John N. Lavis

**Affiliations:** 1 McMaster Health Forum, McMaster University, 1280 Main Street West MML-417, Hamilton, ON L8S 4L6 Canada; 2 Health Policy PhD Program, McMaster University, 1280 Main Street West, Hamilton, ON L8S 4L6 Canada; 3 Faculty of Social Sciences, McMaster University, 1280 Main St W Kenneth Taylor Hall, Hamilton, ON L8S 4M4 Canada; 4 Department of Health Research Methods Evidence and Impact, McMaster University, 1280 Main Street West, 2C Area, Hamilton, ON L8S 4K1 Canada

**Keywords:** Evidence-informed decision making, Evidence-informed policy, Evidence-based practice, Methodology

## Abstract

**Background:**

Globally, a growing number of calls to formalize and strengthen evidence-support systems have been released, all of which emphasize the importance of evidence-informed decision making. To achieve this, it is critical that evidence producers and decision-makers interact, and that decision-makers’ evidence needs can be efficiently translated into questions to which evidence producers can respond. This paper aims to create a taxonomy of demand-driven questions for use by evidence producers, intermediaries (i.e., people working in between researchers and decision-makers) and decision-makers.

**Methods:**

We conducted a global cross-sectional survey of units providing some type of evidence support at the explicit request of decision-makers. Unit representatives were invited to answer an online questionnaire where they were asked to provide a list of the questions that they have addressed through their evidence-support mechanism. Descriptive analyses were used to analyze the survey responses, while the questions collected from each unit were iteratively analyzed to create a mutually exclusive and collectively exhaustive list of types of questions that can be answered with some form of evidence.

**Results:**

Twenty-nine individuals completed the questionnaire, and more than 250 submitted questions were analysed to create a taxonomy of 41 different types of demand-driven questions. These 41 questions were organized by the goal to be achieved, and the goals were grouped in the four decision-making stages (i) clarifying a societal problem, its causes and potential impacts; (ii) finding and selecting options to address a problem; (iii) implementing or scaling-up an option; and (iv) monitoring implementation and evaluating impacts.

**Conclusion:**

The mutually exclusive and collectively exhaustive list of demand-driven questions will help decision-makers (to ask and prioritize questions), evidence producers (to organize and present their work), and evidence-intermediaries (to connect evidence needs with evidence supply).

**Supplementary Information:**

The online version contains supplementary material available at 10.1186/s12961-024-01160-4.

## Background

Evidence has become a crucial component of decision-making processes and, by supporting decision-makers to address a broad variety of issues, from identifying problems to analysing potential solutions and evaluating the implementation of actions, it can play a significant role in several stages of the policy cycle [1–3].

In recent years, there has been a growing number of calls to coordinate and strengthen the global evidence architecture [4–6]. These calls stem from the recognition that evidence-informed decision making is essential for implementing better programs and policies, and that high-quality evidence is necessary for decision-making.

These calls have also stressed that there is a critical need to match and integrate the different forms of evidence to support the steps and varied needs in the decision-making process, and to further strengthen global evidence architecture. In this paper, we adopt the broad definition of evidence used by the Global Commission on Evidence to Address Societal Challenges [5], which includes all forms of decision-relevant evidence (data analytics, modelling, evaluation, qualitative insights, behavioural/implementation research, evidence syntheses, guidelines, and technology assessments).

Despite these global calls and the momentum created by the COVID-19 pandemic, there remains a continuing risk of mismatch between decision-makers’ needs and the evidence that is made available to support decision-makers [7]. There are several factors that can help to explain why decision-makers’ needs are not always fully addressed by research evidence [8]. One factor is that decision-makers have multiple evidence needs and the types of questions that are traditionally used by researchers are limited in scope [e.g., PICO (population, intervention, comparison, outcome) [9], SPIDER (sample, phenomenon of interest, design, evaluation, research type) [10], and PEO (population, exposure, outcome)].

It is critically important that decision-makers understand what types of question that evidence might usefully address, and that evidence producers and intermediaries (i.e., people working in between researchers and decision-makers) understand how to translate decision-makers’ needs into questions that can be used to address these needs [11]. Such understanding can help to build trust, promote more and better interactions, and increase the usefulness and use of existing evidence.

This paper aims to create a taxonomy of questions that evidence can help to answer. Specifically, it aims to:

1. Create a list of types of questions that decision-makers around the world have commonly asked of those they turn to for decision-relevant evidence.
2. Create a mutually exclusive and collectively exhaustive list of such questions.

## Methods

This study is a cross-sectional survey of evidence-support units providing evidence support to decision-makers. These units provide evidence-related advice to decision makers on a timely and regular manner. The study aims to collect different types of questions that decision-makers regularly ask, to identify the wide range of questions where evidence could provide decision-relevant insights, and to develop a mutually exclusive and collectively exhaustive taxonomy of types of questions. This study was approved by the Hamilton Integrated Research Ethics Board (HiREB), Project ID: 8279.

### Participants

Between March and May 2022, representatives of evidence-support units were invited to answer a questionnaire, which was administered online via a link provided by email to each participant. We understand an evidence-support unit as a group that provides timely, demand-driven summaries of what’s known and not known—based on the best available research evidence—about a question facing decision-making. To be eligible, units needed to:

- Answer questions in response to a request coming from decision-makers, including (but not necessarily limited to) government policymakers (i.e., units addressing real-life evidence needs from decision-makers).
- Address issues that are not exclusively in the clinical domain (for health-focused units).
- Have produced at least five evidence-informed answers in the last 5 years (i.e., the unit is or has recently been active).

Participants that did not produce evidence-support at an explicit request of decision-makers, or that were only focused on clinical answers were excluded from this study.

Representatives of existing evidence-informed policymaking networks, the most recent of these being EVIPNet, were identified and contacted to verify if they were eligible to participate. These representatives were contacted and asked if they were filling the criteria described above to be eligible to participate in this study. Alternatively, they were also be asked if they were aware of other potentially eligible units.

### Data collection

The online questionnaire requested the various types of questions that decision-makers regularly ask the unit and, when possible, for a more complete list of the questions they had previously addressed, a URL link to their products. The questionnaire also collected basic information regarding the scope of the work that each unit performs in supporting decision-making processes. The questionnaire was first piloted with two different centres to assess whether it was easy to complete or that the instructions would need further details.

The questionnaire was sent to participants, and one person per unit was eligible to answer. The questionnaire was originally written in English, but participants were also allowed to answer in French or Spanish if they felt more comfortable answering in those languages. The questionnaire is available in Additional file 1.

### Data analysis

The data collected in the survey were summarised using descriptive analyses and reported with absolute numbers and frequencies. For the questions that were provided by participants, many of them were very similar (e.g., effectiveness of a specific intervention). Hence, for each participant, the 10 most recent questions that each unit reported to have answered were collected aiming at capturing a broad variety of types of question.

Later, these questions were categorized in an iterative thematic analysis to create a mutually exclusive and collectively exhaustive list. If necessary, compound questions answered by these units were split into multiple fundamental questions, and questions were excluded if: (1) they were questins into which evidence cannot provide decision-relevant insights; (2) they were aiming to collect information about what other recommendations have said (e.g., what do scientific societies recommend about a given health condition?); (3) they were explicitly described as having not been asked by a decision-maker; and (4) they were addressed by building on other frameworks (e.g., agenda setting) that do not involve foreground evidence.

The initial draft taxonomy that was created from the responses and structured using the policy cycle framework [12]. In this process, types of questions and goals were created in an inductive way, while the stages were taken from an existing framework (i.e., policy cycle). Additionally, this original draft was complemented by using existing frameworks included in the Evidence Commission report [5], the GRADE Evidence to Decision (EtD) framework [13], and the Consolidated Framework for Implementation Research (CFIR) [14]. Finally, taking advantage of national, regional, and global meetings, a number of people were engaged in deliberations about how to improve the clarity and comprehensiveness of the taxonomy.

## Results

Twenty-seven units were initially identified as potentially eligible, and seven additional units were suggested by participants. Two participants either declined or were found to be ineligible to participate, leaving 32 final potential participants. Twenty-nine answers were received (response rate 90.6%), but only 20 provided a list of questions that could be extracted. In total, 1076 questions were provided. By sampling the 10 most recent questions that were addressed by participants, we analysed a total of 237 different questions.

Table 1 provides details about survey participants. The majority of the units surveyed were based in a university, national ministry, or non-governmental organization. While they accept requests from many types of actors, including government policymakers, managers and program implementers, they most commonly answer requests coming from mid-level policymakers and program implementers. Finally, they serve different domains within the health sector, namely clinical management, public health decisions, health-system (not including technology assessment) decisions and technology assessments.

**Table 1:** Characteristics of units participating on the study

	***N***	**%**
Units currently active in answering decision-making needs		
Type of institution		
University	9	36
National ministry	6	24
Non-governmental organization	5	20
Government agency	3	12
Sub-national ministry	1	4
Other	1	4
Actors that are eligible to request evidence support		
Mid-level policymakers (e.g., head of units, departments)	24	96
High-level policymakers (e.g., ministers, vice-ministers)	22	88
Staff in charge of program implementation	21	84
Managers in government agencies	18	72
People working in NGOs	15	60
People that are part of universities	12	48
Other	4	16
Actors that commonly request evidence support		
Mid-level policymakers (e.g., head of units, departments)	22	88
Staff in charge of program implementation	16	64
People working in NGOs	10	40
High-level policymakers (e.g., ministers, vice-ministers)	9	36
Managers in government agencies	9	36
People that are part of universities	7	28
Other	7	28
Area and sector of work		
Public health	22	88
Health systems (not including technology assessments)	21	84
Clinical practice	12	48
Health technology assessments	9	36
Other	1	4

**Fig. 1:**
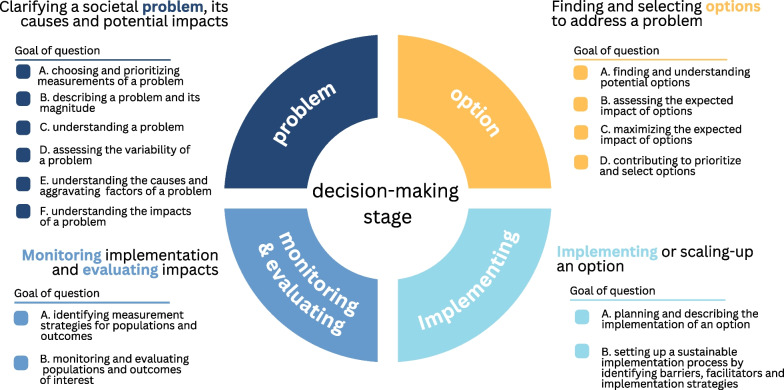
Taxonomy of demand-driven types of questions structured by decision-making stage

Figure 1 shows the goals of each decision-making stage. In total, 41 different types of questions were identified and characterized as part of this taxonomy. To facilitate the understanding of the taxonomy, Tables 2, 3, 4 and 5 describe the types of questions included in each goal. A lay formulation of each goal is also provided in every table, and below. In each decision-making stage, to identify some concepts that are commonly used in certain disciplines to name specific types of questions, notes provide explanations of technical discipline-specific language. Additional file 2 presents a more detailed description of each type of question.

**Table 2:** Goals and types of questions for decision-making stage 1: clarifying a societal problem, its causes and potential impacts

Goals	Types of question
A. Choosing and prioritizing measurements of a problem *Lay language: how can a problem be measured?*	1. Identifying measurements to characterize a problem
	2. Understanding individuals' values regarding outcomes
	3. Prioritizing measurements to characterize a problem
B. Describing a problem and its magnitude *Lay language: what’s the problem and how big it is?*	1. Describing a problem in a point in time
	2. Clarifying and characterizing populations affected by a problem
C. Understanding a problem *Lay language: how and why is a problem?*	1. Finding conceptual approaches to understand a problem
	2. Understanding stakeholders' perceptions of a problem
	3. Understanding the context in which a problem occurs
D. Assessing the variability of a problem *Lay language: how the problem varies over time, across populations and in relation to other problems?*	1. Assessing variability over time
	2. Assessing variability across populations and contexts
	3. Assessing the importance of a problem relative to other problems
E. Understanding the causes and aggravating factors of a problem *Lay language: what is causing or making the problem worse?*	1. Identifying causes and/or aggravating factors of a problem
	2. Understanding the relative importance of causes and/or aggravating factors across populations and contexts
F. Understanding the impacts of a problem *Lay language: what impacts is the problem creating?*	1. Identifying impacts/spillover effects of a problem
	2. Prioritizing the most important impacts/spillover effects of a problem

In epidemiological research, describing a problem through frequencies is often called prevalence (e.g., number of people living with a given health condition) or incidence (e.g., number of people diagnosed with a given health condition during a certain time)

In clinical research, the most common signs and symptoms of a given condition are often called the clinical presentation of a disease

In epidemiological research, causes can also be referred to as risk or protective factors that individuals can experience when they are exposed to a certain cause

In clinical research, the factors that could explain better or worse clinical outcomes on a given health condition are commonly called prognostic factors, while the potential causes of a health condition are called the aetiology of the disease

In public health research, some potential factors that could explain different health outcomes are called (social) determinants of health

In some social sciences field, they could also be understood as explanatory factors, to understand what social factors would cause a given social behaviour

In economics, the unintended impacts of a given action are called externalities (e.g., passive smoking)

**Table 3:** Goals and types of questions for decision-making stage 2: finding and selecting options to address a problem

Goals	Types of question
A. Finding and understanding potential options *Lay language: what are the potential solutions?*	1. Scoping a list of potential options
	2. Understanding the way potential options and their components work
B. Assessing the expected impact or antecedents of options *Lay language: is it feasible (can it work), does it work, is it convenient, and is it equitable and acceptable?*	1. Assessing the feasibility of an option
	2. Assessing the benefits and early and frequently occurring harms of an option
	3. Identifying late-occurring harms of an option
	4. Assessing the acceptability of an option
	5. Assessing the costs and resource use of an option
	6. Assessing the efficiency in the use of resources
	7. Identifying equity, ethical, social and human rights impact of an option
C. Maximizing the expected impact of options *Lay language: how can we ensure success with these solutions?*	1. Adjusting options and enabling factors to maximize impact
	2. Finding population groups and contexts to focusing options
D. Contributing to prioritize and select options *Lay language: how to prioritize or combine solutions?*	1. Creating packages of options
	2. Creating a ranking of options

### Stage 1. Clarifying a societal problem, its causes, and potential impacts

This stage aims to clarify a problem, identify potential causes, and outline potential impacts or spillover effects that this problem might create. It is organized into six different goals that may need to be achieved (A to F). In total, this stage includes 15 different types of questions that may need to be answered (Table 2).

Although ‘problems’ create a decision-making scenario that frames an issue in a negative way, an issue can also be framed in a positive way as objectives (or once a problem has been identified, it can also be framed positively as objective). Then, the goals included in this section can also be framed in a positive or more neutral way by replacing problems by objectives, such as: A. Choosing and prioritizing measurements to determine whether an objective has been reached; B. Describing an objective and its implications; C. Understanding an objective; D. Assessing variability of an objective and its implications; E. understanding the preliminary steps and critical opportunities to reach out an objective; and F. Understanding the impacts of achieving an objective. We will continue by describing this stage as a ‘problem’ assuming that, as mentioned here, the question can be easily formulated using neutral or positive rhetoric.

Problems may be issues that are in the present or the past, but they can also be issues that are not necessarily a problem now, but that could eventually become one (future problems, including existential risk). These future problems were not created as specific types of questions, acknowledging that the same types of questions that are included in this stage can be equally formulated for future problems.

Problems can also arise from issues created in other decision-making stages (e.g., no feasible option is available, an implementation strategy does not address a barrier, or the option has not had the impact that it should have had, or its impact failed to be sustained). In these cases, users of this taxonomy might consider the issue as a new problem and identify a question that could match this issue in this decision-making stage.

Questions related to people’s values and experiences (e.g., values regarding outcomes, understanding people’s perceptions, etc.) might also vary according to some social characteristics, such as socioeconomic status, ethnicity, etc., and these issues are somehow included in these types of questions.

### Stage 2. Finding and selecting options to address a problem

This stage aims to find and select options that could address (or help to reduce) the impact of a problem. It is structured as four distinct goals that may need to be achieved (A to D). In total, this stage includes 13 different types of questions that may need to be answered (Table 3).

Similar to problems, options can be present or past interventions, or they can also be interventions that are not available right now but could become an option in the future. Specific questions for these issues were not created, acknowledging that the same types of questions that are included in this stage can be formulated for present for future options.

The types of question included here are in the context of options not yet implemented and it is their possible impact that is assessed. The actual impact of the implementation of an option in decision-making will be addressed in stage 4 (Monitoring implementation and evaluating impacts).

Identifying the equity, ethical and human rights implications of an option could be understood as whether the impact of the option had different implications depending on specific population characteristics (e.g., socioeconomic status, ethnicity, etc.).

### Stage 3. Implementing or scaling-up an option

This stage aims to address issues related to the implementation of a given option. It is structured around two different goals that may need to be achieved (A and B). In total, this stage includes 6 different types of questions that may need to be answered (Table 4).

**Table 4:** Goals and types of questions for decision-making stage 3: implementing or scaling-up an option

Goals	Type of questions
A. Planning and describing the implementation of an option *Lay language: can it be done and what needs to happen to implement?*	1. Identifying who has to do what to implement an option
	2. Identifying the context in which the option could be implemented
	3. Describing the extent and stage level to which implementation is underway
B. Setting up a sustainable implementation process by identifying barriers, facilitators and implementation strategies *Lay language: how can implementation be improved?*	1. Identifying and understanding barriers and facilitators to implement and option
	2. Identifying and understanding implementation strategies to deal or take advantage of barriers and facilitators
	3. Prioritizing barriers, facilitators and implementation strategies

In implementation sciences, options (or interventions) can also be called innovations or change management tools

In implementation sciences, the implementation process could also be called scale and spread

Implementing an option is a critical stage in the decision-making process. However, there are some interventions in which the implementation stage might not necessarily be critical (e.g., prescribing a clinical treatment course for a given hospitalized patient).

The conditions that an option requires to be implemented can be classified using behavioural (e.g., what individuals need to do for the option to be implemented) and/or contextual (that are often split in relevant to the inner and outer settings) variables. The contextual variables, and the setting (i.e., inner and/or outer setting), include the potential equity implications that the implementation of a given option might have.

### Stage 4. Monitoring implementation and evaluating the impacts of an option or implementation strategy

This stage aims to monitor the implementation of a given option and to evaluate its causal impacts in a particular setting. It is structured as two different goals (A and B). This stage includes 7 different types of questions that may need to be answered (Table 5). Monitoring implementation and evaluating impacts can be done at the short, medium and/or long-term; identifying measurement strategies for problems and options are also a key part of this stage.

**Table 5:** Goals and types of questions for decision-making stage 4: monitoring implementation and evaluating the impacts of an option or implementation strategy

Goals	Types of question
A. Identifying measurement strategies for populations and outcomes *Lay language: how can we measure populations and results?*	1. Identifying instruments to identify or categorize populations
	2. Choosing the most accurate instruments to identify or categorize populations
	3. Identifying measurement instruments for outcomes of interest
	4. Determining the best instruments to measure outcomes of interest
B. Monitoring and evaluating populations and outcomes of interests *Lay language: has it achieved what it was supposed to achieve?*	1. Monitoring the implementation of an option or implementation strategy
	2. Evaluating the impact of an option or implementation strategy
	3. Interpreting the findings of monitoring implementation or evaluating the impact of an option or implementation strategy

Several frameworks build on evidence coming from this type of question to better understand the impact of a given intervention (e.g., theory of change, logical framework, etc.) and its mechanism of action

## Discussion

### Principal findings and findings in relation to the existing literature

This paper develops a taxonomy of mutually exclusive and collectively exhaustive types of demand-driven questions in which evidence may provide decision-relevant insight. We identified forty different types of questions, which were classified across 14 different goals in four different decision-making stages. Some existing frameworks have been developed to formulate research questions, such as PICO [9] and SPIDER [10], or to understand what type of categories or typologies of research questions can be addressed by evidence syntheses [15, 16], or facilitating models for the taxonomy of research studies[17]. However, these frameworks were not built with a demand-driven approach (complemented by existing frameworks as the one presented in this paper) to facilitate decision-making.

Although the field of knowledge translation has substantially evolved in recent decades, knowledge translation efforts and tools have concentrated on how new research findings can be better disseminated to decision-makers [18]. However, no available tools facilitate the interaction between decision-makers and evidence producers or intermediaries (i.e., people working in between researchers and decision-makers) at the question-formulation stage to achieve a more responsive evidence-support system.

A recently renewed focus on the co-production of knowledge—understood as a collaboration between evidence producers, decision makers, and any other stakeholder to design, implement and interpret research for a given need [19]—has of course yielded outputs that can support the future flow of new research. This taxonomy provides a more actionable output, which could be used to help in co-produce evidence support. Hence, when a decision-making need emerges, collaborative work among decision-makers, evidence intermediaries and evidence producers facilitated by the taxonomy created in this paper might make easier to clarify the specific question for which an evidence-informed answer is required.

### Strengths and limitations

This study has several strengths. First, this is the first paper that creates a mutually exclusive and collectively exhaustive list of types of question for which evidence could provide decision-relevant support. Secondly, the taxonomy was created using a demand-driven perspective by asking evidence-support groups to itemize the questions they have received from decision-makers. Hence, it is built from existing questions that have been addressed by at least one of a variety of operating evidence-support units. Finally, it uses generic language that facilitates the communication across different sectors/disciplines and different forms of evidence.

This study has also some limitations. First, it was infeasible to reach all the units that provide some type of support across all sectors and disciplines, and participants working in non-surveyed sectors might provide extensions to this taxonomy, which can affect the representativeness of the study population. Also, while this paper presents a mutually exclusive and collectively exhaustive list of types of question, it has not yet been applied to a specific setting or context to validate and facilitate the understanding of this taxonomy. Finally, despite the units that participated in this study provided demand-driven support, the questions received by them were the ones that they answered, which might not necessarily be the ones that they were requested to answer.

### Implications for policy and practice

This taxonomy can have different implications depending on three main audiences. First, decision-makers (including government policymakers, professionals and citizens) could easily scan the different types of questions to clarify the type of questions for which evidence could provide decision support. Second, impact-oriented evidence producers of any form of evidence could better orient their work to organize and prioritize types of questions, enhancing coordination and avoiding duplication among them. Finally, this tool could strongly support evidence-intermediaries in connecting the demand needs with the supply side.

When using this taxonomy of types of question, users should bear in mind the following considerations. First, although we have presented the types of question in a logical order, they are by no means intended as a list each of which those making policy decisions should consider for each one of their issues. Indeed, decision-makers can use one, some, or all of the questions to address a given issue. By providing guidance on what questions from this taxonomy would most usefully be addressed to answer a specific decision or specific fields, evidence intermediaries could facilitate this selection.

Secondly, some types of question included might not be relevant for certain groups (e.g., comparing the importance of a problem against others in social sciences, or prioritizing spill over effects across different sectors). Thirdly, our aim in developing the taxonomy was to organize questions and not the results that research answering these questions could have. Hence, since they are essentially an assessment of the answer of a specific type of question, we considered questions such as “What are the evidence gaps or the methodological limitations of the existing evidence for a given topic?” out of the scope. Finally, there are several types of question that are addressed by building on other complex frameworks (e.g., agenda setting of a policy issue [20]; chances of a policy to be developed looking at institutions, interests and ideas [21] or the political economy; or the external validity of a given body of evidence). These questions are important, and several types of questions from the taxonomy could contribute to conducting an assessment in these complex frameworks.

### Implications for future research

This taxonomy of research questions is only a first of many efforts that could facilitate the connection between demand-side needs and evidence production and support. Further research should explore how different study designs could properly answer each type of question identified in this taxonomy. A concrete application of this taxonomy in a case study would help to validate and test the tool. Matching types of decisions (e.g., funding a new technology, what intervention to use for addressing a specific problem, whether acting now is the right time, conducting or not a pilot for a new technology) with the types of questions included in this taxonomy would, by specifying what types of question in this taxonomy should be answered depending on the specific type of decision, facilitate a stronger and more integrated evidence-support system.

Future research efforts could also go back to the survey participants and interviewing: (1) a sample of them to ask whether they have encountered additional questions that were not represented in the taxonomy, because they have been addressed by complementary groups in other sectors, or in groups that provide a more integrated evidence-support to decision-makers in a given country; and (2) other actors (e.g., government policymakers, science advisors, subject-matter advisors, etc.) who could provide additional types of question that were not necessarily addressed by evidence advice.

Finally, future uses of the taxonomy in combination with artificial intelligence could consider these types of questions in their algorithms and quickly identify claims that are, or are not, supported by evidence.

## Conclusions

This paper provides a unique taxonomy of 41 demand-driven types of questions where evidence could provide decision-relevant insights, structured around four decision-making stages (clarifying a societal problem, its causes and potential impacts; finding and selecting options to address a problem; implementing or scaling-up an option; and monitoring implementation and evaluating impacts). Decision-makers, evidence intermediaries, and impact-oriented evidence producers could importantly benefit from this taxonomy to facilitate the exchange of evidence needs from decision-makers, through evidence intermediaries and to better connect evidence-production efforts among evidence producers.

### Supplementary Information

Additional file 1. Questionnaire used to collect data from participants.

Additional file 2. Details of the types of question included in the taxonomy.

## Data Availability

The anonymized datasets used during the current study are available from the corresponding author on reasonable request.
