# Supplementary material for: A taxonomy of demand-driven questions for use by evidence producers, intermediaries and decision-makers: results from a cross-sectional survey
Source: Health Res Policy Syst. 2024 Jul 5;22:78. doi: 10.1186/s12961-024-01160-4 (PMC11225415; doi:10.1186/s12961-024-01160-4)
Supplement: Supplementary file 1 — Additional file 1. Questionnaire used to collect data from participants. [file 12961_2024_1160_MOESM1_ESM.docx]

Additional file 1. Questionnaire used to collect data from participants.

**Identifying and categorizing health-policy research questions**

**Online survey**

Questionnaire to be sent to units supporting the use of research evidence in decision making.

| **#** | **Question** | **Question type** | **Possible answers** |
| --- | --- | --- | --- |
| 1 | Is your unit active in answering policymaking needs with research evidence? | Yes/no question | Yes or no |
| 2 | Please select the category that best describes the institution in which your unit is embedded. | Answer one option | National ministry  Sub-national (e.g., provincial) ministry  Government agency  Legislative branch  Judicial branch  Non-governmental organization  University  Other (please specify) |
| 3 | Who is eligible to request evidence support to your unit? | Answer one or multiple options from a list | High-level policymakers (e.g., ministers, vice ministers)  Mid-range policymakers (e.g., heads of units, departments)  Managers of government agencies  Staff in charge of program implementation  People that are part of universities  People working in NGOs.  Other (Please specify) |
| 4 | Who most commonly request evidence support to your unit? | Answer one or multiple options from a list | High-level policymakers (e.g., ministers, vice ministers)  Mid-range policymakers (e.g., heads of units, departments)  Managers of government agencies  Staff in charge of program implementation  People that are part of universities  People working in NGOs.  Other (Please specify) |
| 5 | If you work in the health sector, which area(s) does your unit provide evidence support? | Answer one or multiple options from a list | Clinical practice  Public health [including epidemiology]  Technology assessments  Health systems  Other (please specify) |
| 6 | If available, please provide the full list of research questions that your unit has addressed. | Free text | Free text |
| 7 | If possible, please provide the full list (with hyperlinks if available) of publications or reports produced to inform policymaking processes. | Free text | Free text |
