# Supplementary material for: A taxonomy of demand-driven questions for use by evidence producers, intermediaries and decision-makers: results from a cross-sectional survey
Source: Health Res Policy Syst. 2024 Jul 5;22:78. doi: 10.1186/s12961-024-01160-4 (PMC11225415; doi:10.1186/s12961-024-01160-4)
Supplement: Supplementary file 2 — Additional file 2. Details of the types of question included in the taxonomy. [file 12961_2024_1160_MOESM2_ESM.docx]

Additional file 2. Details of the types of question included in the taxonomy.

**STAGE I. CLARIFYING A SOCIETAL PROBLEM, ITS CAUSES, AND POTENTIAL IMPACTS**

This stage aims to clarify a given problem, identify potential causes, and outline potential impacts or spillover effects of it. It is structured by six different goals that may need to be achieved (A to F). In total, 15 different types of questions that may need to be answered to achieve the goal are included in this stage.

I.A. Choosing and prioritizing measurements of a problem (i.e., how can a problem be measured?)

This goal aims to get insights on what potential outcomes exist to characterize or measure a problem, the different values that individuals could have regarding the outcomes, and to prioritize what are the most suitable outcomes to characterize or measure the problem. Three types of questions are included in this goal:

I.A.1. Identifying measurements to characterize a problem.

Identifying measurements is a critical step of clarifying a problem. This type of question looks for different outcomes that are available and suitable to characterize or measure a given problem.

I.A.2. Understanding individuals' values regarding outcomes

People could have different values on the uses of different outcomes to characterize or measure a problem. Also, different stakeholders could have different values regarding outcomes (e.g., citizens may value different the outcomes than government policymakers).

I.A.3. Prioritizing measurements to characterize a problem.

The prioritization of measurements to characterize or measure a problem can be done by looking at the identified outcomes and weighting the different values and preferences of individuals regarding them.

I.B. Describing a problem and its magnitude (i.e., what’s the problem and how big it is?)

This goal aims to describe a problem in a given moment of time and to identify the population that is affected by it. Two types of questions are included in this goal, namely.

I.B.1. Describing a problem in a point in time

The description of a problem or objective in a given moment of time can be done depending on the type of variable that would need to be measured. Hence, variables can be depicted, among other forms, using the following forms:

Frequencies (e.g., what is the (cumulated or new) frequency of a given characteristic within a population group?)

Central tendencies (e.g., what is the mean of a given characteristic within a population group?)

Distributions (e.g., what is the variation range of a given characteristic within a population group?)

By describing a problem using any of the mentioned variables, the magnitude of the problem is also presented and assessed.

Note about jargon: In epidemiological research, describing a problem through frequencies is often called **prevalence** (for number of people living with certain characteristics in a given moment of time) or **incidence** (for new number of people that started to have certain characteristics during a certain time). In clinical research, the frequency of signs and symptoms of a given condition is also called the **clinical presentation** of a disease.

I.B.2. Clarifying and characterizing populations affected by a problem.

To clarify a problem, the population that is affected by it might not always be clear. This type of question aims to specify the population and specific sub-populations that are affected (or most affected) by a certain problem.

I.C. Understanding a problem (i.e., how, and why is a problem?)

This goal aims to interpret a given problem by critically and conceptually analyzing it and understanding how it is perceived by different stakeholders and the role that the context has in the specific problem. Three types of questions are included in this goal:

I.C.1. Finding conceptual approaches to understand a problem.

Depending on the complexity of a given problem, conceptual frameworks might be useful to understand a problem, which might lead to better ways on how to address it. This question includes finding and selecting frameworks to describe and conceptualize a complex problem or issue, and how the problem is created by interconnecting with other factors (e.g., behavioral, and contextual variables).

I.C.2. Understanding stakeholders' perceptions of a problem

Exploring the perceptions that different groups have of a problem is critical to understand the problem and its magnitude. There are many types of stakeholders that might have different perceptions of the same issue, including government policymakers, organizational leaders, professionals, and citizens.

The interpretations of the results of a study answering this type of question can be used to understand the different framing of a given problem by a certain interest group.

Note about jargon: An important economic branch studies the role of **stated and revealed preferences** to distinguish between stakeholders’ perceptions that are communicated (‘stated') versus the ones that are not evidently communicated (‘revealed’).

I.C.3. Understanding the context in which a problem occurs.

This question includes the understanding of the role that the context or the specific setting has in the problem. Contexts and/or settings can bring unique challenges/opportunities for a certain problem or objective to be developed or achieved.

I.D. Assessing the variability of a problem (i.e., how the problem varies over time, across populations and other problems?)

This goal aims to assess the variability or magnitude of a given problem comparing against time, other populations or settings, and other problems. The variability can be assessed by comparing only one variable (i.e., either time, other populations, or other problems) or multivariable (e.g., against time and other populations at the simultaneously). Three types of questions are included in this goal:

I.D.1. Assessing variability over time

Assessing the variability of a problem or issue when compared against time could be done over limited (e.g., 2 points) or multiple (e.g., time series) point of time. This type of question measures the evolution of an indicator or measurement over time.

I.D.2. Assessing variability across populations or settings

Assessing the variability of a problem or issue when compared against other populations or settings aims to explore how the problem is in other populations or settings. This type of question includes to identify what populations are most affected by the problem, or what populations are most at risk, which entails equity considerations.

I.D.3. Assessing the importance of a problem relative to other problems

Assessing the variability of a problem when compared against other problems entail to measure the relative importance (i.e., weight) of an issue compared to others.

Note about jargon: In epidemiological research, measuring the burden that a disease can cause compared to other is often called the **burden of disease**.

I.E. Understanding the causes and aggravating factors of a problem (i.e., what is causing or making the problem worse?)

In this goal, the problem is set as the dependant variable (being caused by something else) as opposed to goal F, where the problem is set as the independent variable (the problem being the cause of something else). However, in many cases, the framing of the question could be unclear (i.e., whether an issue is the problem or the cause of a different problem).

Note about jargon: In epidemiological research, causes can also be referred as **risk or protective factors** that individuals can experience when they are **exposed** to a certain cause. In public health research, some potential factors that could explain different health outcomes are called **determinants of health**. In clinical research, the factors that could explain better or worse clinical outcomes on a given health condition are commonly called **prognostic factors**, while the potential causes of a health condition are called the **etiology of the disease**. In some social sciences field, they could also be understood as **explanatory factors**, to understand what social factors would cause a given social behaviour.

This goal aims to identify and understand the relative importance of causes and/or aggravating factors of the problem. Two types of questions are included in this goal:

I.E.,1. Identifying causes and/or aggravating factors of a problem.

This question asks whether and to what extent a certain variable can be identified as a cause or aggravating factor of a certain problem. Identifying potential causes or aggravating factors of a problem require assessing whether a factor has potentially some effect on a certain outcome at different complexity levels. Different type of causes or aggravating factors might have variable effects on certain outcomes, and the level of exposure to a certain factor might also vary the effects on these outcomes. Finally, causes and aggravating factors might be of several types such as personal characteristics (e.g., age), contexts (e.g., living in rural areas), interventions received (e.g., mechanical ventilation), or

I.E.,2. Understanding the relative importance of causes and/or aggravating factors across population groups and contexts.

After having identified potential causes or aggravating factors of a problem, this type of question assesses the relative importance that causes and/or aggravating factors have based on their contribution to the problem, and to identify in what population groups the association between these causes and/or aggravating factors and the outcome is stronger.

I.F. Understanding the impacts of a problem (i.e., what impacts is the problem creating?)

As mentioned in goal I.C, in this goal the problem is set as an independent variable (the problem being the cause of something else), as opposed to goal I.F, where the problem is set as the dependent variable (the problem being caused by something else). A problem can create several types of impacts or spillover effects, that could be felt in one or multiple sectors (e.g., one health problem could create spillover effects on the environment).

Note about jargon: In economics, the unintended impacts of a given action are called **externalities** (e.g., passive smoking).

This goal aims to identify and prioritize the most important impacts or spillover effects of a given problem. Two types of questions are included in this goal:

I.F.1. Identifying impacts/spillover effects of a problem

This question asks whether and to what extent a given scenario is a consequence of a certain problem. Identifying potential impacts or spillover effects require assessing whether the problem has potentially some effect on a certain outcome at different complexity levels.

I.F.2. Prioritizing the most important impacts/spillover effects of a problem

After having identified potential impacts or spillover effects of a problem, this type of question assesses what are the most important of them, and to identify in what population groups would be most affected by these impacts.

**STAGE 2. FINDING AND SELECTING OPTIONS TO ADDRESS A PROBLEM**

This stage aims to find and select options that could address (or help to reduce the impact) of a problem. It is structured in four different goals that may need to be achieved (A to D). In total, 13 different types of questions that may need to be answered to achieve the goal are included in this stage.

II.A. Finding ad understanding potential options (i.e., what are the potential solutions?)

This goal aims to first identify a list of potential options to address a given problem, followed by understanding how and why they work. Two types of questions are included in this goal:

II.A.1. Scoping a list of potential options

A list of potentially available interventions to address a given problem should be the main output of answering this question. This type of question commonly includes a judgement of what options are potentially suitable for a specific contextual reality and asking what others (including what other jurisdictions) are doing to deal with a given problem.

Interventions might also not be available right now, but they might become available in future (e.g., vaccines under phase I-II trials).

Note about jargon: In clinical research, these potential options would be called **treatment alternatives** for treating a given health condition.

II.A.2. Understanding the way potential options and their components work

This type of question describes what does entail to develop a given option, including its mechanism of action (or causal pathway if applicable), and how and why it should work to address the problem or issue.

An important distinction should be made with an option that has been already implemented, and someone may want to know why it has not had the results that should have had. This issue will be re-taken in the stage 4 of this taxonomy (Monitoring implementation and evaluating impact).

II.B. Assessing the expected impact or antecedents of options (i.e., is it feasible, should it work, is it convenient, and is it equitable and acceptable?)

This goal aims to assess the possible impact or success of options by assessing it in different outcomes. The impact of a given option can be assessed at one point in time, and can be assessed in the short, medium, and long-term to better assess the sustainability of the option.

This goal is sitting in the context in which an option has not yet been implemented, so we are assessing the possible impact of options. We will come back to the actual impact of the implementation of an option in stage 4 (Monitoring implementation and evaluating impact).

Also, the impact of an option can be assessed in different populations, so to well-formulate a question in this stage, someone should have a clear population in which the option is planning to be implemented.

Seven types of questions are included in this goal:

II.B.1. Assessing the feasibility of an option

Assessing this question entails making a judgement whether a given option is it going to be feasible to be implemented in a given context or setting, and it could be split in different dimensions (e.g., operationally feasible, legally feasible, etc.).

II.B.2. Assessing the benefits and early and frequently occurring harms of an option

An option can have benefits to address a problem or its causes. Assessing the benefits entails understanding the outcomes that need to be used to measure benefits, the effect size, and its variability.

Note about jargon: In epidemiological research, the benefits would be called **efficacy** (if it is measured under controlled circumstances) or **effectiveness** (if it is measured under ‘real-world’ circumstances).

II.B.3. Identifying late-occurring harms of an option

An option can also potentially have harms. Identifying them and assessing its probability of occurrence (i.e., risk) are a critical part of assessing the expected impact of options. In case the benefits of an option are also available, this type of question might entail assessing whether the benefits outweigh the harms (i.e., net benefit).

Harms can also be direct from the implementation of a given option or could also be unintentional or be more spillover effects of the implementation of a given option in other sectors or contexts.

Note about jargon: In health economics and health technology assessment, the outcome of assessing whether the benefits of a technology outweigh the harms is called **benefit-risk balance**.

II.B.4. Assessing the acceptability of an option

This type of question measures the level of acceptability that a given option would have in a concrete setting, and to what extent a given group is willing to receive an intervention.

Note about jargon: In some research disciplines, individuals’ **satisfaction** with an option could also be included in this type of question.

II.B.5. Assessing the costs and resource use of an option

The potential costs and resource use that implementing a given option will create depend on the specific context and setting in which the option is planned to be implemented. Resources could be monetary (i.e., costs), but could also be human resources, technology, etc.

Note about jargon: In health economics and health technology assessment, calculating the costs that implementing a new technology will bring to the system is called **budget impact analysis**.

II.B.6. Assessing the efficiency in the use of resources

Using resources to implement an option creates questions related to how efficient these investments are. In this question, we compare whether the costs of implementing an option might be worth it.

Note about jargon: In economics, this efficiency in the use of resources is often called **value for money**. If a previous investment is conducted, **return on investment (ROI)** could also be used. In health economics, the **cost-effectiveness,** and the **incremental cost-effectiveness (cost-utility) ratio** of an intervention is often used to measure the efficiency in the use of resources.

II.B.7. Identifying equity, ethical and human rights impact of an option

Implementing an option might have several equity, ethical and human rights implications. In one side, implementing an option might have differential impact (measured as any of the other outcomes included in this goal) in some population groups. In the other side, some options could have ethical, social, and human rights implications when implemented that might arise when finding and selecting options. This type of question also includes the social value that a given option might have.

II.C. Maximizing the expected impact of options (i.e., how can we ensure success with these solutions?)

This goal aims to maximize the expected impact by either adjusting some variables of interventions, or by focusing the option on certain population groups or settings. Two types of questions are included in this goal:

II.C.1. Adjusting options and enabling factors to maximize impact.

This type of question evaluates whether adjusting some variables (e.g., the deliverer, the intensity of the intervention, etc.) could modify the expected impact (measured as any of the questions provided in II.B) of an option.

Note about jargon: In epidemiological research, variables that modify the impact of an intervention are called **modifiers**.

II.C.2. Finding population groups, settings, and contexts to focusing options

This type of question explores what setting or socioecological contexts, and/or in what population groups the intervention would produce most impact (measured as any of the questions provided in II.B, which includes in what population the intervention would achieve most equitable results). This includes understanding why the impact of an option is different in one context compared to another.

Note about jargon: In social sciences, this type of question would also include the analysis of **positive deviance**, that aims to understand why a reduced number of cases are producing positive results when others are not.

II.D. Contributing to prioritize and select options (i.e., how to prioritize or combine solutions?)

This goal aims to produce insights to select the best combination of options to address the problem or causes, by creating packages or creating a ranking of options.

It is important to notice that selecting what options to pursue would be out of the scope of this list, since many other non-evidence related factors could be considered to make a decision on what to implement, but this goal concentrated on the insights that evidence could provide to these specific types of decisions.

Two types of questions are included in this goal. They are not mutually exclusive, and, in fact, the ranking created could be a ranking of packages.

II.D.1. Creating packages of options

This type of question finds the right combination of interventions that would produce the optimal balance between the expected impacts (using any or a combination of the impacts described in II.B). It can also be framed in a way to ask what are the packages or options that produce a minimal threshold of impact.

II.D.2. Creating ranking of options

This type of question creates a ranking of options (or packages of options) sorted by the expected impact that they would produce (measured as any or a combination of the impacts described in II.B).

This type of question includes inquiries that are looking for the most impactful (e.g., most effective, least harmful) intervention to address a given problem.

**STAGE 3. IMPLEMENTING OR SCALING-UP AN OPTION**

This stage aims to address issues related to the implementation of a given option. It is structured in two different goals that may need to be achieved (A and B). In total, 6 different types of questions that may need to be answered to achieve the goal are included in this stage.

Note about jargon: In implementation sciences, options (or interventions) can also be called **innovations** or **change management tools**.

III.A. Planning and describing the implementation of an option (i.e., can it be done and what needs to happen to implement?)

This goal aims to plan and describe the implementation of a given option by identifying who has to do what to implement an option, what role the context has in the implementation process and what is the implementation level of a given option.

This goal looks at variables and conditions required for a given option to be implemented. These can be structured in behavioral (e.g., what individuals need to do for the option to be implemented) and contextual variables (that are often split in inner and outer settings). This can also be interpreted as what conditions are needed for an option to be feasible to be implemented.

Assuming that one potential decision of a new option might be to conduct a pilot or a implementing an option at small-scale, this goal also aims to draw on lessons learned from the early implementation of a given option to plan the scale-up of it.

Note about jargon: In implementation sciences, the implementation process could also be called **scale and spread.**

Three types of questions are included in this goal:

III.A.1. Identifying who has to do what to implement an option.

This question is looking at finding variables related to stakeholders’ behaviors (behavioral variables) that are required for an option to be implemented (i.e., what people need to do in order to implement an option).

In order to answer this question, the identification of key stakeholders are one step to be conducted before.

III.A.2. Identifying the context in which the option could be implemented.

Many times, the context influences the chances of an option to be implemented. This question is looking at finding variables related to the inner or outer setting or context in which an option is feasible to be implemented (including the political climate).

III.A.3. Describing the extent and stage level to which implementation is underway.

Here we assess the level of implementation of an option (or group of options) in a given moment of time (or over time) in a concrete setting. This could also include how many population groups have been reached by the option over time, and the historical development of a given option in a given jurisdiction.

III.B. Setting up a sustainable implementation process by identifying barriers, facilitators, and implementation strategies (i.e., how the implementation can be improved?)

This goal aims to find barriers and facilitators, and implementation strategies that could address and take advantage of them, respectively. Barriers and facilitators can come from different domains (inner and outer settings, individuals (e.g., skills and capacities), etc.), from different levels (e.g., government, policy, system, etc.), and across different type of actors (e.g., service providers, users or patients, organizations, etc.).

It is not always clear whether a given variable constitutes a barrier or facilitator, or to whether a given variable could be framed in a positive (facilitator) or negative (barrier) way. Then, we can also call barriers and facilitators as implementation considerations.

The implementation process entails identifying strategies to ensure a sustainable implementation of an option. This means to apply implementation strategies that can take the form of interventions to address or take advantage of barriers and facilitators, or they could also be mitigation measures for the potential risks of implementing an option (e.g., risk-control or risk-management strategies), in the short, medium, and long-term.

Three types of questions are included in this goal:

III.B.1. Identifying and understanding barriers and facilitators to implement and option.

Barriers are variables that could block or delay the implementation of a given option. Facilitators are variables that could make the implementation of a given option easier. This question includes the identification of them as well as understanding why they are barriers and why the barriers could interfere or facilitate the implementation of an option.

III.B.2. Identifying and understanding implementation strategies to deal or take advantage of barriers and facilitators.

Implementation strategies to take advantage of them could accelerate the implementation process. This question includes the identification of them as well as understanding why they could work to take advantage of these facilitators.

III.B.3. Prioritizing barriers, facilitators, and implementation strategies.

Once barriers and facilitators have been identified, the assessment of the importance of several barriers and/or facilitators in the implementation of a given intervention in a specific setting is a critical step. Barriers and facilitators could be prioritized in order to reduce the scope and focus an implementation plan. At the same time, implementation strategies (i.e., interventions that could address barriers and take advantage of facilitators) could also be prioritized.

**STAGE 4. MONITORING IMPLEMENTATION AND EVALUATING THE IMPACT OF OPTIONS OR IMPLEMENTATION STRATEGIES**

This stage aims to monitor the implementation of a given option and to evaluate the impact of a given option in a concrete setting. It is structured in two different goals that may need to be achieved (A and B). In total, 7 different types of questions that may need to be answered to achieve the goal are included in this stage.

IV.A. Identifying measurement strategies for populations and outcomes (i.e., how can we measure populations and results?)

This goal aims to identify and select measurement strategies for ascertain the right population and accurately measure the outcomes of interest. This is related to finding instruments to facilitate measurement in order to monitor implementation and evaluate impact. In ascertaining populations and measuring outcomes, the questions are split in first identifying available instruments, followed by the selection of the most suitable instrument. Four types of questions are included in this goal:

IV.A.1. Identifying instruments to identify or categorize populations.

Several measurement instruments might exist to identify the right population to monitor the implementation and/or evaluate the impact of an intervention.

IV.A.2. Choosing the most accurate instruments to identify or categorize populations.

After having identified potential instruments to identify the right population, this question evaluates the accuracy to identify them.

Note about jargon: In clinical research, this is used in the context of the identification of the population with a certain health condition, and it is called **diagnostic accuracy**.

IV.A.3. Identifying measurement instruments for outcomes of interest

Outcomes can be used to measure a problem or an option and can be used to measure in one or multiple points in time. This type of questions identifies the instruments available to measure outcomes.

IV.A.4. Determining the best instruments to measure outcomes of interest.

This question identifies how accurate a given instrument (e.g., measurement or scale) is to measure a given outcome of interest. This can be particularly helpful where there are complex phenomena to measure and where scales acting as proxys might be needed.

Note about jargon: In epidemiological research, this question is often split in **reliability** (the consistency in which a measurement brings the same results) and **validity** (the level of bias that a measurement could have).

IV.B. Monitoring and evaluating populations and outcomes of interests (i.e., has it achieved what it was supposed to achieve?)

This goal aims to monitor and evaluate populations and options or implementation strategies, including its sustainability over time. Here, we focus on scenarios where an option has been already implemented (as opposed to the questions included in stage 2). Two types of questions are included in this goal:

IV.B.1. Monitoring the implementation of an option or implementation strategy

In this type of question, the implementation of an option is monitored to see what the progress is. Here, the question is related to assess whether the option or implementation strategy is achieving the outputs that is planning to achieve. This is sometimes done as monitoring systems to measure whether target indicators have been reached in a given time-frame, or whether the implementation of an option has been feasible (e.g., formative or process evaluation). Similarly, the option or implementation strategy can be monitored using results or process indicators.

IV.B.2. Evaluating the impact of an option or implementation strategy

Measuring the impact could be conducted by using any of the outcomes outlined in the question 2.B. This includes the assessment of potential unanticipated harms (or spillover effects) of an option or implementation strategy. In this type of question, the impact that an option actually had is measured.

IV.B.3. Interpreting the findings of monitoring implementation or evaluating the impact of an option or implementation strategy

This question includes asking why the option or implementation strategy is having the results that is showing and comparing them with the expected impact. It also includes the understanding of the pathway (and intermediate outcomes) for a given option or implementation strategy to produce impact, and to draw lessons learned in this process.

Note about jargon: Several frameworks build on evidence coming from this type of question to better understand the impact of a given intervention (e.g., **theory of change, logical framework**, etc.) and its mechanism of action.
